# An updated review on the role of extracellular vesicles in immune system modulation in breast cancer with special emphasis on immune checkpoint regulators

**DOI:** 10.3389/fimmu.2026.1872143

**Published:** 2026-07-16

**Authors:** Jayenth Jayachandran, Surajit Pathak, Zulya Maizetova, Arunkumar Radhakrishnan, Antara Banerjee, Asim K Duttaroy

**Affiliations:** 1 Department of Pharmacology, Chettinad Hospital and Research Institute (CHRI), Chettinad Academy of Research and Education (CARE), Chennai, India; 2 Medical Biotechnology Lab, Faculty of Allied Health Sciences, Chettinad Academy of Research and Education (CARE), Chettinad Hospital and Research Institute (CHRI), Chennai, India; 3 Cell Lab 7, Masdar City, Abu Dhabi, United Arab Emirates; 4 Department of Nutrition, Institute of Medical Sciences, Faculty of Medicine, University of Oslo, Oslo, Norway

**Keywords:** breast cancer, EVs, exosomes, immune checkpoints, tumor microenvironment

## Abstract

Breast cancer progression and resistance to therapy are strongly influenced by immune evasion within the tumor microenvironment. Immune checkpoint signaling is a major mechanism by which cancer cells evade immune surveillance, thereby promoting tumor progression and reducing the effectiveness of immunotherapy. Recent evidence suggests that extracellular vesicles (EVs) are important mediators of communication between tumor, stromal, and immune cells, enabling the transfer of proteins, nucleic acids, lipids, and other bioactive molecules that regulate immune responses. This review discusses current knowledge on the role of EVs in immune checkpoint regulation in breast cancer, with an emphasis on both programmed death-ligand 1 (PD-L1)-dependent and additional immunosuppressive pathways that collectively contribute to immune escape. A literature review was conducted using PubMed, Google Scholar, and Web of Science, focusing on studies from the past decade related to EV biology, immune checkpoints, and breast cancer. Findings from multiple studies indicate that tumor-derived EVs contribute to immunosuppression by impairing T-cell function, promoting immune tolerance, facilitating metastatic progression, and supporting resistance to immunotherapy. Importantly, EV-mediated effects are different for the breast cancer subtypes, which may play a role in treatment response, disease progression, and clinical outcomes. EVs also show potential as minimally invasive biomarkers for disease monitoring and as therapeutic targets or delivery systems for precision medicine. Overall, this review highlights current evidence on EV-mediated immune checkpoint regulation in breast cancer, highlighting PD-L1 and CTLA-4associated mechanisms as key drivers of immune evasion and promising targets for precision immunotherapy.

## Highlights

- Extracellular vesicles regulate immune checkpoint signaling and drive immune evasion in breast cancer.
- EV-associated PD-L1, CTLA-4, and other immunosuppressive cargos promote T-cell dysfunction and resistance to immunotherapy.

## 1 Introduction

Breast cancer is one of the most commonly diagnosed malignancies in women and remains one of the leading causes of cancer-associated death worldwide (1). Although significant advances have been made in early detection and molecular classification, the global burden of breast cancer continues to increase, particularly in regions where access to screening, early diagnosis, and effective treatment remains limited (2). One of the major challenges to the effective management of breast cancer is its heterogeneity; breast cancer includes a variety of histological types, molecular profiles, clinical behaviors, and different responses to various forms of treatment (3). Furthermore, heterogeneity affects not only disease development and prognosis but also immune evasion and responses to immunotherapy through complex interactions within the tumor microenvironment (4).

The tumor microenvironment (TME) has emerged as a key determinant of breast cancer progression and immune escape (5). The TME is a dynamic and complex system consisting of tumor cells, immune cells, fibroblasts, endothelial cells, adipocytes, and the surrounding extracellular matrix (ECM) (4). Tumor cells interact with the stroma and immune components of the TME, thereby regulating the strength and effectiveness of the anti-tumor immune response (6). Moreover, breast tumors establish an immunosuppressive microenvironment, characterized by regulatory T cells, myeloid-derived suppressor cells, tumor-associated macrophages, and inhibitory cytokines that collectively enable the tumor to evade immune destruction, progress, and become resistant to therapies (7).

Immune checkpoint is a fundamental mechanism by which breast cancer cells evade anti-tumor immunity (8). The primary checkpoints, programmed cell death protein-1 (PD-1) and its ligand programmed death-ligand 1 (PD-L1), CTLA-4, and other related checkpoints/ligands regulate T-cell activation, exhaustion, and immune tolerance in the TME (9). Although immunotherapy has significantly improved clinical results for certain highly immunogenic cancers following the development of immune checkpoint inhibitors, its clinical benefit in breast cancer is still limited. However, clinical results have been found in certain subsets of breast cancer, such as triple-negative breast cancer, due to its high tumor immunogenicity and immune checkpoint protein expression; however, other major subtypes, such as luminal and HER2-positive breast cancers, remain less responsive (10). Poor response rates, immune-mediated toxicities, and acquired resistance underscore an urgent need to discover novel mechanisms that modulate immune checkpoint signaling within the breast cancer TME (11).

Over the past few years, research findings on extracellular vesicles (EVs) have shown that they are important mediators of intercellular communication between tumor cells and their microenvironment (12). EVs are nanoscale membrane-bound vesicles that carry signals derived from tumor and stromal cells. EVs contain a variety of biomolecules, including proteins, lipids, nucleic acids, and metabolites (11). In breast cancer, EVs derived from tumor cells may play a crucial role in remodeling the TME by influencing immune cell activity, enhancing immunosuppressive signaling, and contributing to the establishment of metastatic niches (13). Recent experimental evidence indicates that EVs can influence immune checkpoint regulation by transferring immune checkpoint-associated molecules, including PD-L1, PD-L2, PD-1, CTLA-4, TIM-3, and LAG-3, thereby suppressing T-cell activity and facilitating tumor immune escape (14). Through these processes, EVs facilitate immune evasion and may partially explain the limited and variable responses to immune checkpoint-based therapies in breast cancer (15).

While the role of EVs in cancer biology has been widely described, a clear integration of their mechanistic involvement in immune checkpoint regulation with emerging experimental approaches and clinical application remains insufficiently explored. In particular, the potential of EVs as diagnostic biomarkers, therapeutic targets, and delivery vehicles in the context of immune checkpoint modulation requires further evaluation. Hence, further research in this area is urgently required.

In this review, we summarize the role of extracellular vesicle (EV)-mediated immune checkpoint regulation in breast cancer, with particular emphasis on EV-associated PD-L1 and CTLA-4-related mechanisms based on published research findings. We also discuss EVs’ contribution to immunotherapy resistance in breast cancer. Breast cancer-derived EVs carry immune checkpoint proteins, including PD-L1, PD-L2, PD-1, CTLA-4, TIM-3, and LAG-3, either on their membrane surface or within their cargo. These EV-associated proteins are transferred to recipient immune cells, where they suppress T-cell activation, proliferation, and cytotoxic function, thereby promoting immune evasion, tumor progression, and resistance to immune checkpoint blockade.

## 2 Immunological features of the breast cancer tumor microenvironment

### 2.1 Cellular composition of the breast cancer tumor microenvironment

Breast cancer has a complex microenvironment comprising cancer cells, stromal cells, and infiltrating immune cells (15). These interactions are mediated through cytokines, chemokines, and cell-cell signaling pathways that collectively regulate immune activation or suppression within the TME. Recent studies, including those in triple-negative breast cancer (TNBC), have shown that variations in immune and stromal cell composition are correlated with differences in outcome and treatment response. However, similar subtype-specific variations are also observed in luminal and HER2-positive breast cancers, highlighting the broader heterogeneity of the TME across breast cancer subtypes (13).

Within the breast cancer TME, Cancer-associated fibroblasts (CAFs) represent the dominant stromal component and form distinct subpopulations with specialized functions in ECM remodeling, cytokine secretion, and immune regulation (16). Recent single-cell analyses in TNBC identify ECM-rich CAFs that are enriched in non-responders to neoadjuvant immunotherapy and that create a fibrotic, T cell-excluded niche through SPP1^+^ macrophage-driven TGF-β/IL-1β signaling (17). Furthermore, CAF subsets actively recruit and modulate tumor-associated macrophages (TAMs), myeloid-derived suppressor cells (MDSCs), and regulatory T cells (Tregs), thereby establishing a multilayered immunosuppressive network that limits effective anti-tumor immunity (5).

Myeloid cells, particularly tumor-associated macrophages (TAMs) and MDSCs, are increasingly recognized as major drivers of immunosuppression within the breast cancer TME (18). Recent studies in TNBC report that TAM-enriched tumors exhibit reduced T-cell infiltration and poorer clinical outcomes, while blockade of CSF1R can reprogram or deplete TAMs, thereby enhancing the efficacy of low-dose cyclophosphamide and inducing robust T helper and B cell-mediated immune responses with sustained tumor regression (19). Mechanistically, TAMs contribute to immune evasion through the secretion of immunosuppressive cytokines (e.g., IL-10 and TGF-β), expression of immune checkpoint ligands including PD-L1 and PD-L2, and metabolic reprogramming that suppresses T-cell activity. In parallel, multi-omics analyses of TNBC tumors have identified distinct immune programs with prognostic significance, such as IL32^+^ regulatory T cells and MDSC-like myeloid clusters, which are consistently associated with immunotherapy resistance (20).

The lymphoid compartment also displays marked heterogeneity across breast cancer subtypes, as TNBCs with high tumor-infiltrating lymphocyte levels show strong CD8^+^ T-cell and natural killer cell cytotoxic signatures and improved responses to chemo-immunotherapy, whereas many hormone receptor-positive tumors exhibit low T-cell infiltration and poor responsiveness to immune checkpoint therapy (21). This subtype-specific immune landscape suggests that differences in antigen presentation, cytokine signaling, and immune cell recruitment contribute to variability in therapeutic outcomes. A recent study integrating malignant cell-intrinsic gene signatures, such as UQCRFS1-based indices, with TME composition further demonstrated that tumor genomics and the immune system are functionally interconnected and jointly influence patient survival and immunotherapeutic outcomes across multiple cancer types (13, 22).

### 2.2 Immune checkpoint landscape in breast cancer

The PD-1/PD-L1 axis remains the principal target of immunotherapy in breast cancer, with patients with TNBC deriving the most consistent clinical benefit (23). However, both primary and acquired resistance are frequently observed. Emerging evidence indicates that resistance to PD-1/PD-L1 blockade arises through multiple interconnected mechanisms, including the loss of tumor antigenicity, irreversible T-cell exhaustion, dysfunction of the interferon-γ signaling pathway, the accumulation of suppressive myeloid cells, and overexpression of alternative immune checkpoints (24). In addition, recent analyses of cancers and breast cancer have further highlighted the role of impaired antigen presentation, low tumor-infiltrating lymphocyte density, oncogenic signaling pathways, and epigenetic changes in the development of immune resistance, which together account for a subset of patients being resistant to therapy (25, 26).

Recent findings indicate that chemotherapy-resistant tumor cells may also overexpress PD-L1 (CD274) or other ligands, such as CD80, particularly in senescent tumor cells, thereby ensuring continued immune evasion even after successful initial treatment (27). Recent experimental studies using TNBC cell lines demonstrate that PD-1/PD-L1 blockade can lead to compensatory overexpression of other immune checkpoints, including CTLA-4, TIM-3, and LAG-3, on CD4^+^ T-cell subsets, highlighting checkpoint switching as a mechanism of resistance (28).

Importantly, immune checkpoint expression is highly heterogeneous across breast cancer subtypes. Molecules such as PD-1, CTLA-4, TIM-3, LAG-3, TIGIT, and VISTA exhibit variable expression patterns across molecular subtypes, tumor grades, and immune infiltration status (29). While TNBC generally exhibits higher checkpoint expression and immune infiltration, luminal and HER2-positive subtypes demonstrate distinct checkpoint profiles that may influence their response to immunotherapy. This variability suggests the need for subtype-specific therapeutic strategies rather than a uniform immunotherapeutic approach.

**Figure 1:**
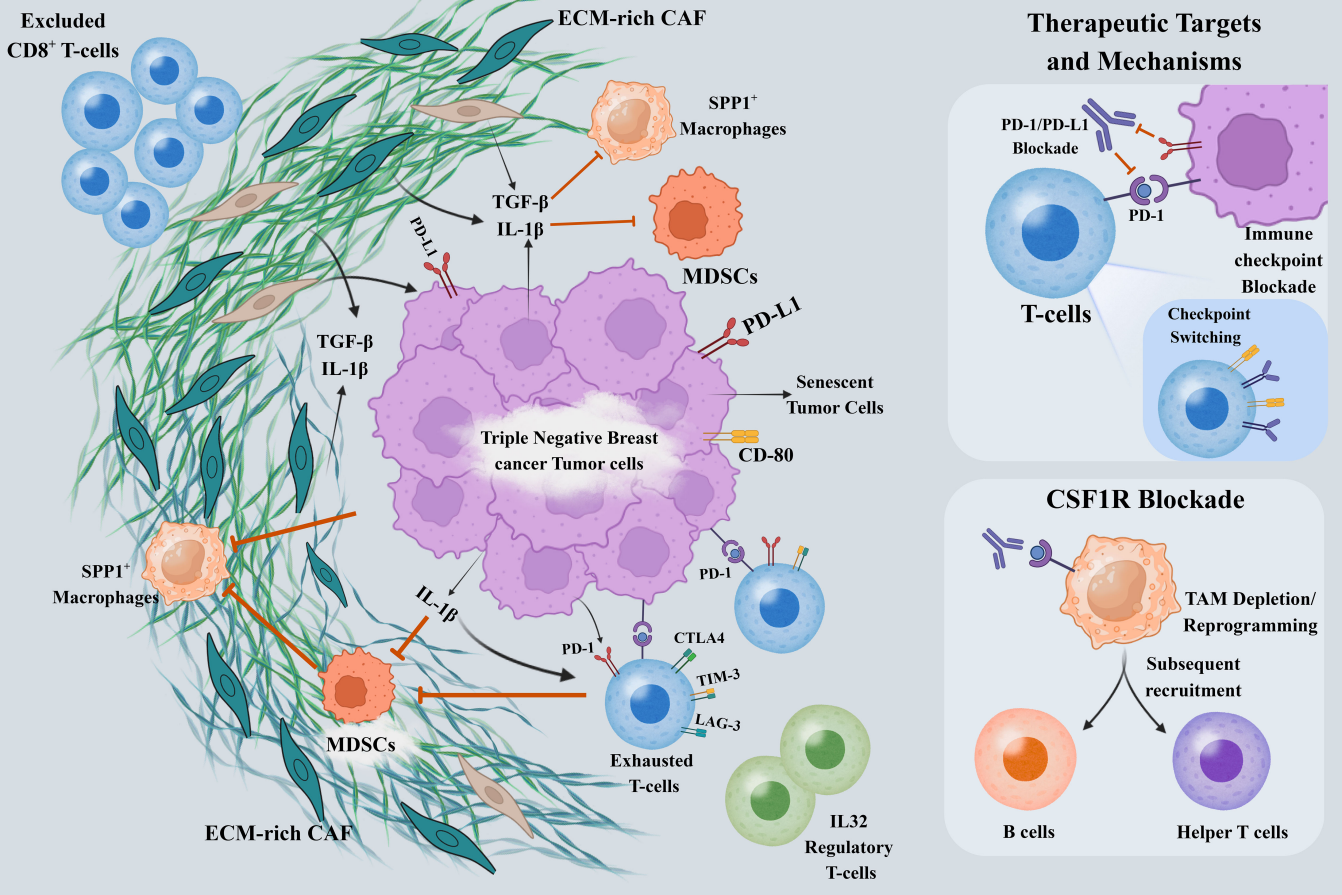
Immunosuppressive cellular landscape and therapeutic targets within the triple-negative breast cancer tumor microenvironment.

Recent studies have identified TIGIT and VISTA as emerging therapeutic targets. TIGIT inhibition has been shown to restore cytotoxic T lymphocyte and NK cell activity, whereas VISTA expression on tumor-associated macrophages and breast cancer cells is associated with aggressive disease and immune suppression (30). However, most of these findings remain at the preclinical or early clinical stage, highlighting the need for further validation in large-scale clinical studies. Thus, current evidence supports the development of combination therapeutic strategies targeting PD-1/PD-L1 alongside other immune checkpoints, chemotherapy, and TME-modulating agents to overcome immune resistance (23, 24, 29). The complex interplay among CAFs, myeloid cells, lymphoid populations, and immune checkpoint signaling pathways, which collectively drive immune suppression and therapeutic resistance, is illustrated in **Figure 1**, conceptually developed based on findings reported in references 23-29.

## 3 Extracellular vesicles: biogenesis, composition, and biological functions

EVs have emerged as fundamental mediators of intercellular communication, acting not only as nanoscale carriers of bioactive molecules but also as active regulators of immune signaling within the TME (31). In breast cancer, recent studies show that tumor and stromal cells release markedly increased numbers of EVs that reflect oncogenic and metabolic signaling and microenvironmental stress, thereby promoting tumor growth, immune escape, and metastatic niche formation (32). Recent proteomic and genomic studies demonstrate that breast cancer-derived EVs carry selective cargo associated with angiogenesis, immune modulation, invasion, and metastasis, linking EV composition with disease progression and tumor aggressiveness (33).

Tumor-derived EVs transport diverse cargos, including proteins, lipids, DNA, and non-coding RNAs, that reprogram immune cells such as T cells, NK cells, macrophages, and dendritic cells toward immunosuppressive phenotypes and support angiogenesis, epithelial-mesenchymal transition, and resistance to anticancer therapies (34, 35). However, the functional contribution of specific EV cargo components remains context-dependent, and their relative importance *in vivo* conditions is still under investigation, highlighting the need for more mechanistic and quantitative studies.

EVs are broadly classified into exosomes, microvesicles, and apoptotic bodies based on their biogenesis and size characteristics (36). Exosomes are formed after the fusion of multivesicular bodies with the cell membrane (37), whereas microvesicles form through the outward budding of the plasma membrane (38), and apoptotic bodies arise during programmed cell death (39). Due to overlapping size and characteristics among EV subtypes, the collective term “EVs” is often recommended for clarity. In breast cancer, functional differences among EV subpopulations are increasingly evident, as proteomic analyses of EVs from cell lines such as MCF-7, MDA-MB-231, and T47D reveal enrichment of proteins associated with angiogenesis, immune modulation, invasion, and metastasis, thereby correlating EV cargo composition with tumor aggressiveness (33). Additionally, organotropic mouse models demonstrate that distinct EV protein signatures, including integrins, correspond to metastatic organ tropism, underscoring the role of specific EV populations in modulating metastatic conditions (40). Despite these advances, standardization in EV isolation and characterization remains a significant challenge, limiting cross-study comparability. The major classes of EVs, their biogenesis pathways, size ranges, and key immunoregulatory cargo relevant to breast cancer are summarized in **Table 1**.

**Table 1:** Types of EVs, biogenesis, and immunoregulatory cargo in breast cancer.

Types of EVs	Size range	Biogenesis pathway	Key immunoregulatory cargo	Functional relevance in breast cancer TME	References
Exosomes (small EVs)	~30–150 nm	Endosomal pathway: intraluminal vesicle formation within multivesicular bodies followed by fusion with the plasma membrane.	PD-L1, MHC-I/II, TGF-β, integrins, miR-21, miR-155, miR-1246, lncRNAs, metabolic enzymes	Suppress CD8^+^ T-cell activation, induce T-cell exhaustion, promote Treg stability, macrophage M2 polarization, systemic immune suppression, and therapy resistance	(31–36)
Microvesicles (ectosomes)	~100–1000 nm	Direct ectosomal release from the plasma membrane.	PD-L1, cytokines, phosphatidylserine, proteases	Local immune suppression, modulation of antigen presentation, and stromal remodelling	(36–38)
Apoptotic bodies	~500–5000 nm	Plasma membrane fragmentation during apoptosis.	Nuclear fragments, DNA, histones, immunomodulatory proteins	Immune tolerance induction, antigen dissemination, and potential pro-inflammatory or tolerogenic effects depending on context	(39)

Importantly, EV-mediated signaling varies across molecular breast cancer subtypes, reflecting subtype-specific oncogenic pathways and immune landscapes (41). In luminal breast cancer, EVs are primarily associated with endocrine resistance through the transfer of regulatory miRNAs and metabolic mediators that alter estrogen receptor signaling (42). In HER2-positive breast cancer, EVs contribute to therapeutic resistance through HER2 protein transfer, modulation of trastuzumab response, and regulation of immune checkpoint signaling (43). In contrast, in TNBC, EVs play a dominant role in immune modulation, macrophage polarization, PD-L1 signaling, and metastatic niche formation, consistent with the aggressive and immunologically active nature of this subtype (44). These subtype-specific differences highlight the need for tailored EV-targeted therapeutic strategies rather than a uniform approach across breast cancer types.

### 3.1 Release of extracellular vesicles in breast cancer

EV biogenesis and release in cancer cells are tightly regulated by endosomal and plasma membrane dynamics, with cancer progression influencing these regulatory mechanisms (45). EV transport occurs through ESCRT (endosomal sorting complex required for transport)-dependent and ESCRT-independent pathways, with the ESCRT machinery playing a central role in exosome biogenesis (46). This process involves selective cargo sorting, inward membrane budding, and maturation of multi-vesicular bodies (47). These processes are coordinated through interactions between cytoskeletal elements, membrane lipids, and intracellular signaling pathways, linking EV biogenesis to cellular stress responses and oncogenic signaling (48).

Breast cancer cells dynamically regulate EV secretion, particularly under hypoxic and oncogenic conditions (49). Hypoxia alters the expression of proteins involved in EV trafficking and secretion, leading to increased EV production (50), also enriching EVs with pro-metastatic and immunosuppressive components (51). Mechanistically, hypoxia-inducible factors (HIFs) influence endosomal sorting and vesicle release, thereby connecting environmental stress to EV-mediated immune modulation and metastatic progression (46).

The functional impact of breast cancer-derived EVs is largely driven by their selectively loaded molecular cargo, including proteins, nucleic acids, lipids, and metabolites, which reprogram neighboring immune and stromal cells within the TME (52). Proteomic analyses demonstrate enrichment of immune checkpoint molecules, adhesion proteins, angiogenic factors, and metabolic enzymes within EVs, supporting their role in intercellular signaling (41, 53). In addition to PD-L1, EVs have been reported to carry other immunoregulatory proteins such as Fas ligand (FasL), transforming growth factor-β (TGF-β), V-domain Ig suppressor of T-cell activation (VISTA), and ligands associated with LAG-3 signaling including fibrinogen-like protein 1 (FGL1) and Galectin-3, which collectively contribute to T-cell suppression, immune tolerance, and tumor immune escape (54, 55). Notably, EV-associated PD-L1 can suppress T-cell activity both directly and indirectly by modulating antigen-presenting cells, including macrophages and dendritic cells (56, 57).

In addition to immune checkpoint ligands, EVs carrying metabolic regulators (e.g., glucose transporter 1 (GLUT1), pyruvate kinase M2 (PKM2), lactate dehydrogenase A (LDHA), and miR-122) and integrins (e.g., integrin α6β4, α6β1, and αvβ5) play significant roles in extracellular matrix remodeling and in resistance to therapies (58, 59). EVs also carry regulatory non-coding RNAs, including miRNAs, lincRNAs, and circRNAs, that modulate gene expression in recipient cells (32). For example, EV-mediated transfer of miR-21, miR-155, and miR-1246 has been shown to reprogram macrophages toward immunosuppressive phenotypes by reducing antigen presentation and increasing immune checkpoint ligand expression (60). However, the parallel functions and context-specific effects of EV-associated RNAs remain incompletely understood, necessitating further validation in *in vivo* models and clinical samples.

Beyond proteins and RNAs, EV-associated bioactive lipids and metabolites influence vesicle stability, membrane fusion, metabolic reprogramming, immune synapse formation, and inflammatory signaling (61, 62). Collectively, these findings position EVs as highly specialized mediators of oncogenic and immunomodulatory signaling; however, translating these insights into clinical applications remains challenging due to limitations in EV isolation, targeting specificity, and large-scale validation (63, 64). Emerging evidence highlights the potential of EV-associated molecules, particularly EV-derived non-coding RNAs (e.g., miR-21, miR-155, miR-222, and lncRNA H19) and checkpoint ligands (e.g., PD-L1 and CD47), as diagnostic biomarkers and therapeutic targets. Nevertheless, further studies are required to establish the clinical utility, sensitivity, and specificity of EV-based biomarkers and therapeutic strategies in breast cancer immunotherapy (31, 65).

## 4 EV-mediated immune checkpoint regulation in breast cancer

Immune checkpoint regulation within the breast cancer TME is increasingly recognized as a dynamic and spatially coordinated process that extends beyond tumor cell intrinsic signaling (63). EVs serve as active mediators of checkpoint signaling, transporting immune checkpoint proteins and regulatory molecules across the tumor, stromal, and immune compartments (60). Their ability to mediate long-range intercellular communication enables EVs to propagate immunosuppressive signals beyond the primary tumor site, thereby contributing to immune tolerance, T-cell dysfunction, and resistance to immunotherapy (66). Importantly, EV-mediated checkpoint regulation involves multiple inhibitory pathways rather than a single dominant axis, providing a broader mechanistic perspective of tumor immune evasion. Beyond PD-L1, EVs can modulate CTLA-4, TIM-3, LAG-3, TIGIT, and CD47-associated signaling either directly or indirectly, creating a network of complementary immunosuppressive mechanisms. This coordinated checkpoint crosstalk enhances immune escape, contributes to therapeutic resistance, and highlights the need for multi-targeted immunotherapeutic strategies.

### 4.1 Extracellular vesicles-associated PD-L1 and immune suppression

Among immune checkpoint molecules, PD-L1 is the most extensively characterized EV-associated regulator of immunosuppression (67). Breast cancer cells actively package PD-L1 into small EVs, utilizing intracellular routes mediated by the TME, including tumor-generated signals, low oxygen, and inflammation (60). EV-associated PD-L1 retains functional activity, enabling it to bind PD-1 receptors on immune cells even in the absence of direct tumor-immune cell contact (68).

EV-associated PD-L1 inhibits T-cell receptor signaling by disrupting downstream activation pathways, thereby reducing T-cell proliferation, cytokine secretion, and cytotoxic activity (69). Chronic exposure to EV-PD-L1 promotes T-cell exhaustion, characterized by diminished effector function and increased expression of additional inhibitory receptors (70). Unlike membrane-bound PD-L1, EV-associated PD-L1 can circulate systemically, reaching tumor-draining lymph nodes and distant immune sites, thereby amplifying immune suppression beyond the local TME (71). Clinically, elevated levels of circulating EV-PD-L1 correlate with aggressive disease, metastasis, and poor response to immune checkpoint blockade (70). Moreover, EV-PD-L1 contributes to both intrinsic and acquired resistance by maintaining inhibitory signaling even when tumor surface PD-L1 is therapeutically blocked (72). However, the quantitative contribution of EV-PD-L1 relative to tumor cell–associated PD-L1 remains incompletely defined, highlighting a key area for future investigation.

Beyond PD-L1, EVs are also known to transport additional immune regulatory proteins that contribute to immune evasion in breast cancer (44). For example, EV-associated TGF-β can suppress cytotoxic T-cell and NK-cell activity while promoting regulatory T-cell differentiation (73). Similarly, EV-associated Fas ligand (FasL) and galectin family proteins have been implicated in the induction of T-cell apoptosis and immune exhaustion (69). Although these pathways are less well characterized than PD-L1, their combined activity suggests that EV-mediated immunosuppression is multifactorial and cannot be effectively targeted by single-axis blockade alone. While PD-L1 remains the most extensively studied and clinically targeted checkpoint, emerging evidence suggests that other EV-associated inhibitory pathways, including TGF-β, FasL, and alternative checkpoint ligands, contribute to immune evasion through complementary mechanisms. Compared to PD-L1, these pathways are less well characterized but may play context-dependent roles, highlighting the need for combinatorial targeting strategies to achieve effective immunotherapeutic responses.

### 4.2 Extracellular vesicles modulating T-cell function

In addition to direct checkpoint signaling, breast cancer-derived EVs modulate T-cell biology through multiple complementary mechanisms (74). Exposure of cytotoxic CD8^+^ T cells to tumor-derived EVs results in reduced cytotoxicity, decreased interferon-γ production, and impaired clonal expansion (63). These effects are mediated not only by checkpoint ligands such as PD-L1 but also by EV-associated non-coding RNAs and metabolic regulators that reprogram intracellular signaling pathways critical for T-cell function (75).

EVs also promote the development and stability of regulatory T-cells (Tregs) in the TME (75). EV cargo influences transcriptional and metabolic programs that enhance Treg differentiation and suppressive capacity, thereby shifting the balance from effector to regulatory immune responses (76). This imbalance between CD8^+^ T cells and Tregs represents a key mechanism underlying immune tolerance in breast cancer (63). Furthermore, EVs influence T-cell metabolism, which is essential for maintaining effector function and survival (77). EV-mediated transfer of regulatory RNAs and metabolites alters glucose and lipid metabolism in T cells, promoting metabolic exhaustion and reducing their ability to mount effective anti-tumor responses (63). This metabolic reprogramming is closely linked to increased checkpoint signaling and reduced responsiveness to immunotherapy, further reinforcing immune resistance (25).

### 4.3 Extracellular vesicles-driven modulation of innate immune cells

EVs originating from breast cancer cells also target adaptive immunity and profoundly modulate the components of innate immunity (78). Tumor-derived EVs promote macrophage polarization from a pro-inflammatory M1 phenotype to an immunosuppressive M2 phenotype through the transfer of PD-L1, cytokines, and microRNAs, thereby enhancing angiogenesis, tissue remodeling, and immune suppression (79). This macrophage reprogramming represents a critical link between EV signaling and the establishment of an immunosuppressive TME.

Dendritic cells exposed to breast cancer-derived EVs exhibit impaired antigen uptake, reduced expression of co-stimulatory molecules, and diminished capacity to prime naïve T cells (80). As a result, EVs disrupt the initiation of effective adaptive immune responses, further contributing to immune evasion. Similarly, EVs suppress natural killer (NK) cell activity by downregulating activating receptors and reducing cytotoxic granule release, thereby impairing tumor cell clearance even in the absence of antigen presentation (81).

**Figure 2:**
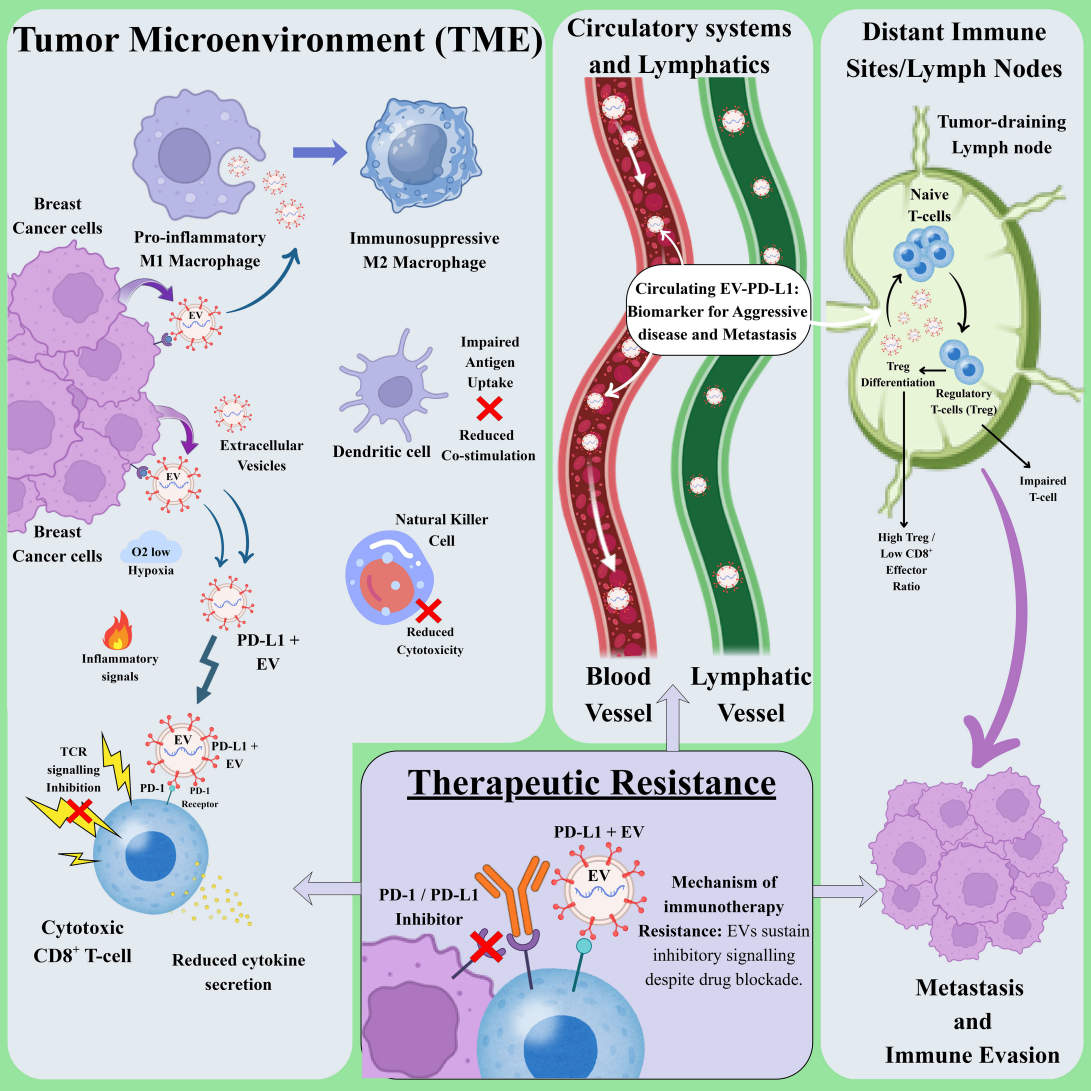
Extracellular vesicle-mediated immune checkpoint regulation, immune cell reprogramming, and therapeutic resistance in breast cancer.

Collectively, these findings demonstrate that EVs coordinate the suppression of both innate and adaptive immune responses through integrated checkpoint signaling, metabolic reprogramming, and cellular re-education. However, most current evidence is derived from preclinical models, and the translational relevance of these mechanisms requires further validation in clinical settings. The spatial and functional roles of breast cancer-derived EVs in immune checkpoint regulation, immune cell reprogramming, and systemic immune evasion are schematically illustrated in **Figure 2**, which was conceptually developed based on the findings reported in references 67-81. Additionally, the diversity of EV sources, checkpoint-associated cargo, and target immune cell populations is summarized in **Table 2**.

**Table 2:** EV-mediated immune checkpoint regulation and immune cell reprogramming in breast cancer.

EV source	Checkpoint/cargo	Target immune cells	Mechanistic effect	Consequence of tumor immunity	References
Tumor-derived EVs	PD-L1	CD8^+^ T cells, CD4^+^ T cells	Inhibits TCR signaling via PD-1 engagement; promotes exhaustion	Reduced cytotoxicity, impaired cytokine secretion, and immune escape	(61–66)
Tumor-derived EVs	miR-21, miR-155, miR-1246	Macrophages, DCs	Downregulates antigen presentation, promotes M2 polarization	Immunosuppressive myeloid landscape	(32, 48, 54)
Tumor-derived EVs	Metabolic enzymes, lipids	T cells	Alters glucose/lipid metabolism; induces metabolic exhaustion	Reduced effector function, poor ICI response	(55–57)
CAF-derived EVs	TGF-β, immunoregulatory ncRNAs	T cells, macrophages, DCs	Enhances checkpoint signaling; suppresses immune infiltration	Immune exclusion, resistance to immunotherapy	(78–80)
TAM-derived EVs	PD-L1, anti-inflammatory mediators	CD8^+^ T cells, NK cells	Reinforces inhibitory signaling; dampens cytotoxic responses	Maintenance of immune tolerance	(82–85)

## 5 Crosstalk between tumor-derived extracellular vesicles and stromal cells

The breast cancer TME is not defined solely by malignant cells and infiltrating immune cells but is regulated by multiple interacting factors, including stromal components that establish physical, biochemical, and immunological barriers to effective antitumor immunity (83). EVs act as key mediators of bidirectional communication between tumor and stromal compartments, facilitating the transfer of oncogenic signals, immunoregulatory molecules, and metabolic factors (84). Through these interactions, EVs regulate stromal remodeling, immunosuppression, and therapeutic resistance, forming an integrated communication network within the TME. Furthermore, EV-mediated communication reprograms stromal cells into tumor-supportive phenotypes, thereby enhancing extracellular matrix remodeling, angiogenesis, and metastatic dissemination. In turn, activated stromal cells release EVs enriched with bioactive cargo that further amplify immune evasion, promote tumor progression, and contribute to resistance against conventional and immune-based therapies.

### 5.1 Extracellular vesicle-mediated regulation of stromal cell subtypes in breast cancer

Breast cancer stromal compartments comprise heterogeneous cell populations that work cooperatively to govern tumor growth, immune tolerance, and chemotherapy resistance (85). Recent evidence indicates that tumor-derived EVs actively reprogram stromal cells, thereby altering their functional roles to support tumor progression (86). Cancer-associated fibroblasts (CAFs), the predominant stromal cell population that influences breast tumors, are significantly regulated by tumor-derived EVs that carry TGFβ-associated signals, miRNAs, and metabolic modulators (87). These EV-mediated signals promote fibroblast activation and drive CAF phenotypes that contribute to extracellular matrix remodeling, T-cell exclusion, and immune suppression (88). Similarly, tumor-derived EVs modulate endothelial functions by transferring pro-angiogenic factors, including VEGF-associated proteins and integrins, as well as hypoxia-related miRNAs, thereby promoting angiogenesis, vascular permeability, and tumor metastasis. The breast tumor EVs have been shown to modulate adipocytic functions by inducing metabolic reprogramming and inflammatory signaling pathways, resulting in the release of fatty acids (89, 90).

Also, these EV-induced MSCs secrete immunosuppressive cytokines and angiogenic mediators and shed tumor-supportive EVs that enhance epithelial-mesenchymal transition, metastasis, and immune escape (91). Emerging evidence further suggests that EVs regulate vascular support cell function and extracellular matrix remodeling, modulating stromal cells, altering vessel stability, and promoting fibrotic niche formation that restricts immune cell infiltration (92). Collectively, these findings demonstrate that tumor-derived EVs establish a coordinated stromal reprogramming network that integrates structural remodeling, metabolic support, and immune regulation. This stromal reprogramming provides the foundation for more specialized EV-mediated interactions discussed in subsequent sections (93). Stromal cell-derived EVs may also transfer immunoregulatory proteins such as TGF-β and galectins, further reinforcing immune checkpoint signaling and immune exclusion within the TME (94).

### 5.2 Cancer-associated fibroblast (CAFs)-derived extracellular vesicles and immune checkpoint activation

Beyond their role in tumor-derived EV-mediated stromal reprogramming, CAFs themselves act as active sources of EVs that further amplify immunosuppressive signaling within the TME. CAF-derived EVs are enriched with immunomodulatory proteins, non-coding RNAs, and metabolic regulators that enhance immune checkpoint pathways and restrict immune cell infiltration (95). These EVs function both locally and systemically to reinforce immunosuppression beyond the tumor-stroma interface. In addition to direct checkpoint modulation, CAF-derived EVs influence immune cell behavior by impairing dendritic cell maturation and limiting effective antigen presentation, thereby reducing T-cell activation. Rather than repeatedly inducing macrophage polarization, CAF-derived EVs primarily act by restricting immune cell recruitment and function through extracellular matrix remodeling and checkpoint signaling pathways. This effect is particularly evident in immune breast cancer subtypes, where increased CAF abundance correlates with poor immunotherapy response (96). Importantly, CAF-derived EV-mediated immune suppression can occur independently of tumor cell-intrinsic checkpoint expression, highlighting a parallel stromal-driven mechanism of immune evasion. This may partly explain the limited efficacy of immune checkpoint therapies in tumors with a strong stromal barrier (97).

### 5.3 Immune cell-derived extracellular vesicles in breast cancer

In addition to tumor-stroma interactions, immune cells within the TME also contribute to EV-mediated communication networks, creating feedback loops that further modulate immune responses (98). Macrophages in tumors, regulatory T-cells, and activated effector T-cells release EVs that modulate the immune system through feedback mechanisms, either augmenting or attenuating antitumor responses. Tumor-associated macrophage-derived EVs are generally immunosuppressive and carry molecules of immune checkpoint ligands, anti-inflammatory mediators, and regulatory RNAs (99). These EVs help maintain an immunosuppressive environment while simultaneously enhancing tumor cell survival and invasion (100).

**Figure 3:**
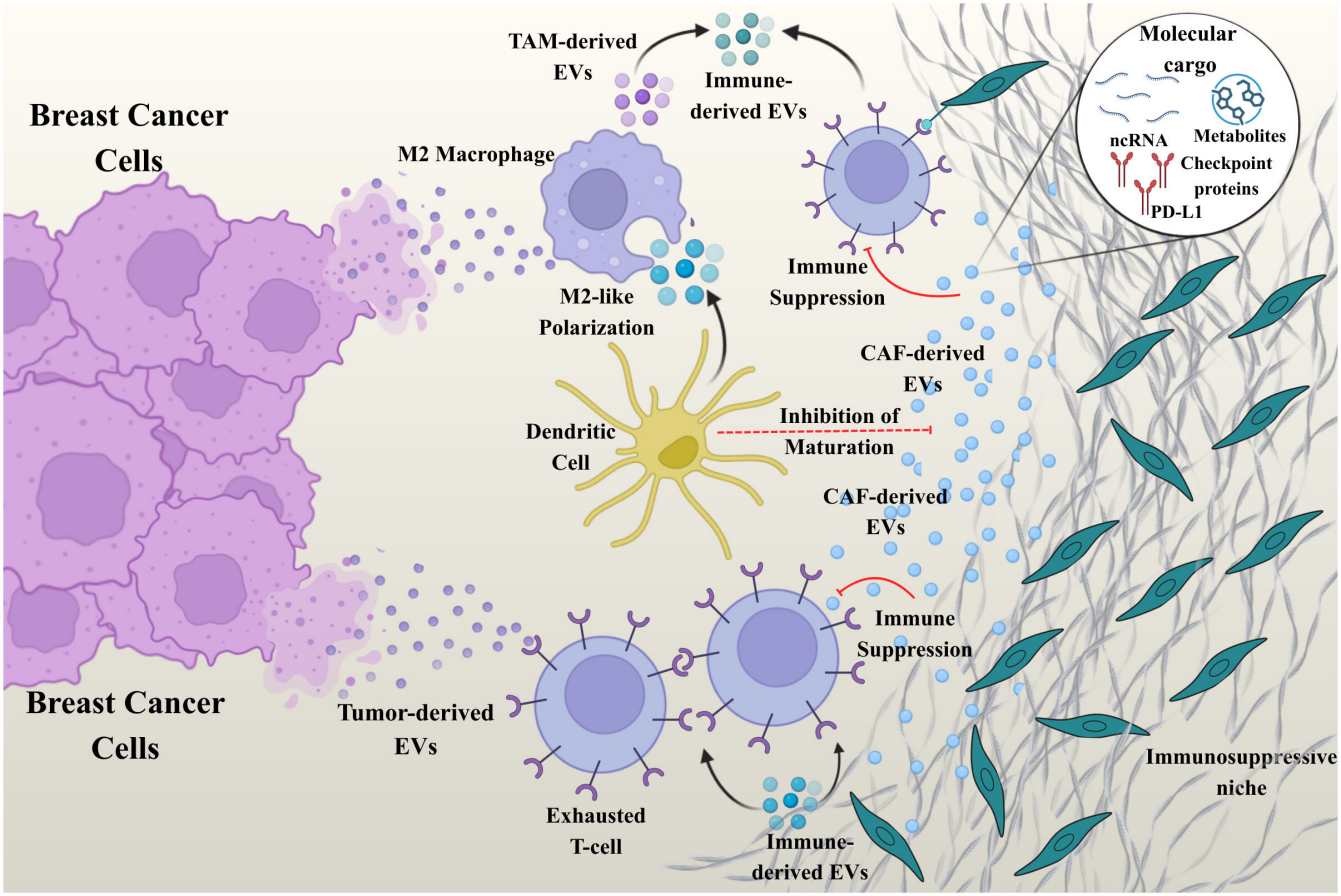
Extracellular vesicle-mediated crosstalk between tumor cells, stromal fibroblasts, and immune cells in the breast cancer tumor microenvironment.

Activated T cells also release EVs containing cytokines, receptors, and regulatory RNAs; however, under chronic tumor microenvironmental conditions characterized by hypoxia, nutrient deprivation, inflammation, and oxidative stress. These EVs can propagate suppressive signaling and reinforce immune exhaustion (101). Thus, immune cell-derived EVs do not uniformly enhance anti-tumor responses but instead participate in a context-dependent regulatory network that often favors immune suppression in breast cancer. Collectively, tumor-, stromal-, and immune cell-derived EVs form an interconnected communication system that reinforces immune checkpoint signaling, stromal remodeling, and metabolic reprogramming. This integrated EV network creates a self-sustaining immunosuppressive microenvironment that promotes tumor progression and resistance to therapy (102, 103). A schematic overview of tumor-stroma-immune cell communication mediated by EVs, including checkpoint ligand transfer and immune cell reprogramming, is shown in **Figure 3** and was conceptually developed based on findings reported in references 85-103.

## 6 Breast cancer extracellular vesicles and resistance to immune checkpoint therapy

Immune checkpoint inhibitors have played a positive role in cancer therapy, although their clinical benefit in breast cancer remains limited and heterogeneous (103). Increasing evidence implicates EVs as central contributors to both intrinsic and acquired resistance to immune checkpoint therapy, operating through systemic, dynamic, and non-genetic mechanisms that extend beyond tumor cell-restricted checkpoint expression (60). One of the most compelling EV-mediated resistance mechanisms involves the immune checkpoint inhibitors being blocked by circulating tumor-derived EVs (87). EVs expressing PD-L1 on their surface can bind therapeutic anti-PD-1 or anti-PD-L1 antibodies, functioning as molecular decoys that reduce drug availability at the tumor-immune interface. This process diminishes the effectiveness of checkpoint blockade, even in tumors that are PD-L1-positive by conventional immunohistochemistry (104). This is because EVs are very common and can pick up and transport immune checkpoint inhibitors both within and outside immune microenvironments, thereby suppressing the collective reactivation of T cells (105).

PD-1/PD-L1-positive EVs are not merely alternative antibody-binding platforms; they may also influence the efficacy of immune checkpoint inhibitors through mechanisms beyond simple antibody capture (106). These circulating vesicles can compete with tumor cells for antibody binding by displaying checkpoint molecules on their surface, potentially reducing the amount of therapeutic antibody that reaches its intended target within the tumor microenvironment (107). Such interactions may alter antibody biodistribution, reduce drug availability, and contribute to persistent immunosuppressive signaling, thereby promoting both intrinsic and acquired resistance to immunotherapy (108). In addition, EV-associated PD-L1 can remain biologically active and interact with PD-1-expressing immune cells, leading to T-cell dysfunction, reduced cytokine production, and impaired anti-tumor immune responses (109). EV-mediated transfer of checkpoint proteins may also extend immunosuppressive signaling beyond the primary tumor site and contribute to immune evasion in distant tissues (68). Furthermore, circulating PD-1/PD-L1-positive EVs have shown promise as potential biomarkers of treatment response and resistance in patients receiving immune checkpoint inhibitors (70, 110).

Despite these findings, several important gaps remain in understanding the functional significance of PD-1/PD-L1-positive EVs. One major challenge is distinguishing vesicle-associated checkpoint molecules from soluble proteins or other extracellular components that may co-isolate during EV purification, thereby affecting the accuracy of EV cargo analysis (68). Moreover, the relative contribution of EV-associated PD-L1 to therapeutic resistance, compared with that of tumor cell-surface PD-L1, remains unclear. Another issue is the heterogeneity of EV populations, as different EV subtypes may differ in cellular origin, checkpoint expression, cargo composition, and biological activity. Consequently, the specific EV populations that drive immunosuppression and treatment resistance remain poorly defined (70). Standardized methods for EV isolation, characterization, and quantification, together with mechanistic and clinical studies, are needed to determine whether PD-1/PD-L1-positive EVs can be reliably used as predictive biomarkers, therapeutic targets, or indicators of resistance to immune checkpoint blockade. Although increasing evidence supports their role in immune regulation and therapeutic resistance, their precise contribution relative to tumor cell-associated checkpoint expression requires further investigation (110).

Moreover, these interactions involve mechanisms far more complex than simple antibody binding, as EV-associated PD-L1 can directly mediate immunosuppression and promote T-cell dysfunction independently of therapeutic intervention (111). The interaction between PD-L1 on EVs and PD-1 on CD8+ T-cells has been shown to suppress T-cell receptor signaling, promote exhaustion, and block the generation of effector/memory T-cells (112). This is especially seen in tumors that produce large numbers of PD-L1-containing EVs from the beginning (113). They could become unresponsive to immune checkpoint treatment since they already live in a suppressive environment (58). They would develop resistance either *de novo* or acquire resistance through treatment, which could stimulate the secretion of increased numbers of EVs and the loading of PD-L1. This could occur through interferon γ- mediated inflammation (114). The immunomodulatory cargo in EVs, including miRNAs, enzymes, and metabolic modulators, reprograms the TME, thereby compromising the efficacy of checkpoint therapy (79). EVs modulate the polarization of macrophages, inhibit antigen presentation of dendritic cells, and suppress cytotoxic T-cell function. When considered collectively, these contribute to a hot tumor transitioning to an immune-excluded (31).

There is growing clinical evidence pointing to EV-mediated mechanisms as significant contributors to immunotherapy response and failure in patients with breast cancer (103). Correlation between elevated concentrations of EV-PD-L1 and poor response rate, progression-free survival, and time to relapse has also been observed with checkpoint inhibitor therapies (70). Decreases in EV-PD-L1 levels reflect immune system activation and successful treatment response, and they stand out as promising non-invasive tools for monitoring response and the development of resistance to therapeutic intervention in the future (115).

## 7 Clinical implications of extracellular vesicle-based immune checkpoint modulation

The emerging role of EVs in immune checkpoint regulation has significant clinical implications in breast cancer, spanning biomarker development, disease monitoring, and therapeutic intervention strategies (116). Unlike conventional tissue-based biomarkers, EVs provide a dynamic and minimally invasive platform for capturing real-time tumor-immune interactions, offering insights into both tumor-intrinsic and microenvironmental immune regulation.

### 7.1 Triple-negative breast cancer extracellular vesicles as diagnostic and prognostic biomarkers

Circulating EVs are emerging as minimally invasive liquid biopsy platforms that may reflect dynamic tumor immune checkpoint activity through the stable transport of checkpoint proteins and regulatory nucleic acids (117). Although many studies have focused on TNBC, emerging evidence indicates that EV-based biomarkers are also relevant across luminal and HER2-positive breast cancer subtypes, reflecting the broader heterogeneity of breast cancer. Circulating EV-PD-L1 levels have been shown to correlate with immune checkpoint activity and clinical outcomes more consistently than tissue PD-L1 in certain contexts, as EVs capture contributions from both tumor cells and the TME (118). However, the predictive value of EV-PD-L1 may differ across subtypes, with stronger associations reported in immunologically active tumors such as TNBC compared to hormone receptor-positive subtypes, which typically exhibit lower immune infiltration.

In addition to protein markers, non-coding RNAs, including miR-21, miR-155, and miR-1246, contribute to immune regulation by influencing T-cell function, macrophage polarization, and therapy resistance (52). Recent evidence suggests that some molecules detected in EV samples may not actually be inside extracellular vesicles. Instead, they may be free biomolecules that were isolated together with the EVs as co-isolates (119). Although many miRNAs actively enter EVs through selective cargo loading mechanisms, some miRNAs may associate with EV surfaces through passive adsorption or interactions with proteins and other biomolecules coating the vesicle surface during circulation and sample processing (120). Differentiating between these compartments is technically challenging, since conventional isolation approaches often co-isolate surface-associated molecules together with the enriched EVs (121). This molecular segregation has important biological implications as encapsulated miRNAs are generally protected from enzymatic degradation and may facilitate intracellular delivery following EV uptake, while surface-associated molecules may preferentially influence receptor-mediated signaling, immune recognition, biodistribution, and cellular targeting (122). Furthermore, uncertainty about cargo localization complicates mechanistic interpretation and may contribute to variability in EV biomarker studies. There is a need for improved isolation strategies and standardized characterization methods to distinguish functional vesicular cargo from externally associated biomolecules (123). Recent studies suggest that these RNA signatures may complement protein-based biomarkers, improving the sensitivity of EV-based diagnostics; however, their specificity and reproducibility across patient cohorts remain to be fully validated. EV-based liquid biopsies also enable longitudinal monitoring of treatment response. Changes in EV-PD-L1 levels and immunoregulatory RNAs during therapy have been proposed as early indicators of treatment response or resistance (117). Despite this promise, standardization of EV isolation methods and threshold values remains a major limitation for clinical implementation (118).

### 7.2 Targeting extracellular vesicles for breast cancer therapy

One strategy involves inhibiting EV biogenesis and release to reduce the dissemination of immunosuppressive cargo (35). Some preclinical studies show that targeting key regulators such as neutral sphingomyelinase, Rab GTPases, and the ESCRT machinery reduces the secretion of PD-L1-containing EVs and partially restores T-cell activity (124). However, these approaches often lack specificity and may disrupt physiological EV functions in normal cells, raising concerns regarding systemic toxicity and off-target effects. Another approach focuses on blocking EV uptake or promoting their clearance from circulation. Some studies suggest that interfering with EV-cell interactions can reduce immune suppression and enhance anti-tumor immunity (35). Nevertheless, the efficiency and selectivity of these strategies *in vivo* remain limited, and their clinical feasibility has yet to be established.

In contrast to EV inhibition, engineered EVs are being explored as therapeutic delivery systems. Preclinical studies have demonstrated that EVs can be loaded with immune checkpoint inhibitors, siRNAs, or immunostimulatory molecules and delivered selectively to the TME (116). For example, EV-based delivery systems have shown improved stability and reduced systemic toxicity compared to antibody-based therapies in experimental models (52). In breast cancer, engineered EVs have been reported to enhance immune activation in otherwise immunologically “cold” tumors (57). Recent advances in EV engineering have further expanded their application beyond passive cargo delivery toward active immunotherapeutic platforms. Owing to their inherent biocompatibility and natural cargo transport properties, EVs are increasingly being explored as biomimetic nanocarriers for tumor antigens, nucleic acids, and immunomodulatory molecules (125). Furthermore, tumor-derived and engineered EVs have demonstrated potential as cell-free vaccine platforms that enhance antigen presentation, promote dendritic cell maturation, and stimulate anti-tumor immune responses. Advances in lipid-based nanovaccine technologies and immune-modified EV systems have further highlighted opportunities to improve therapeutic delivery and overcome immunosuppressive mechanisms within the TME (126). Despite encouraging preclinical findings, the translation of EV-based therapies faces several challenges, including large-scale production, cargo loading efficiency, targeting specificity, and regulatory considerations. Moreover, most current evidence is derived from *in vitro* or animal models, with limited validation in human clinical studies. Thus, while EV-targeted and EV-based therapeutic strategies hold promise, their clinical applicability remains in its early stages. Also, the diagnostic, prognostic, and therapeutic implications of EV-based immune checkpoint modulation in breast cancer are summarized in **Table 3**.

**Table 3:** Clinical implications of EV-based immune checkpoint modulation in breast cancer.

Application	EV-associated marker/strategy	Clinical relevance	Potential advantage	Limitations/challenges	References
Diagnostic biomarker	Circulating EV-PD-L1	Reflects real-time immune suppression and tumor–immune interactions	Non-invasive, dynamic monitoring	Assay standardization, sensitivity	(64)
Prognostic biomarker	EV-associated miRNA signatures (miR-21, miR-155, miR-1246)	Predicts aggressive disease and poor immunotherapy response	Early risk stratification	Inter-patient variability	(48, 54)
Therapy resistance monitoring	Longitudinal EV-PD-L1 levels	Tracks acquired resistance to ICIs	Real-time treatment adaptation	Clinical validation needed	(86)
Therapeutic targeting	Inhibition of EV biogenesis/secretion (nSMase, Rab GTPases, ESCRT)	Reduces the dissemination of immunosuppressive cargo	Synergistic with ICIs	Off-target effects, toxicity	(35)
Drug delivery	Engineered EVs carrying ICIs/siRNA	Targeted modulation of TME	Reduced toxicity, enhanced delivery	Manufacturing and regulatory hurdles	(48, 51)

### 7.3 Human clinical trials studying the role of extracellular vesicles in breast cancer

At present, no ongoing clinical trials directly evaluate EVs as therapeutic immune checkpoint modulators in breast cancer. However, several ongoing observational and translational clinical studies are investigating circulating EVs and exosomes in breast cancer patients (https://clinicaltrials.gov), providing clinically relevant evidence supporting the role of EVs in tumor-immune interactions and immune checkpoint regulation. Ongoing studies are also being conducted to characterize EVs as diagnostic and prognostic biomarkers in patients with breast cancer undergoing conventional therapies. An ongoing study (NCT05831397) is assessing the changes in EV profiles over time in relation to treatment response in patients with breast cancer undergoing neoadjuvant chemotherapy as a means of evaluating EVs as diagnostic and prognostic biomarkers. Another ongoing interventional study (NCT05955521) is investigating exosomes as prognostic and predictive biomarkers in early breast cancer. In this context, observational studies are also underway to characterize the EVs present in the circulation of breast cancer patients (NCT05798338) and to assess the diagnostic potential of particular EV subtypes, such as glycosylated EVs (NCT0541704). Although these studies are not specifically designed to target immune checkpoint pathways, they generate clinically relevant data on EV-associated immune-regulatory cargo, including PD-L1 and non-coding RNAs. This provides an important translational foundation for future EV-based immunotherapeutic strategies.

Furthermore, early-phase trials outside oncology, such as those investigating mesenchymal stromal cell-derived EVs (e.g., NCT06002841), demonstrate the feasibility of EV-based systemic delivery platforms. These studies highlight a critical translational gap between strong preclinical evidence and the current lack of EV-based immunotherapy trials in breast cancer. Overall, ongoing clinical studies establish the feasibility of EV detection, monitoring, and characterization, but dedicated clinical trials targeting EV-mediated immune checkpoint pathways are still needed to translate these findings into therapeutic applications.

## 8 Future perspectives of using extracellular vesicles in breast cancer treatment

Advances in understanding of EV-mediated immune checkpoint regulation are creating new opportunities for precision immunotherapy in breast cancer. Rather than functioning solely as carriers of molecular cargo, EVs are now recognized as active regulators of immune checkpoint signaling, contributing to immune evasion, therapeutic resistance, and tumor progression (57). This evolving understanding has opened new avenues for overcoming the limitations of current immune checkpoint blockade therapies. In particular, the EV-immune checkpoint axis represents a promising therapeutic target that complements conventional checkpoint inhibition strategies (127). Simultaneous targeting of cellular immune checkpoints and EV-mediated immunosuppressive signaling may achieve more durable immune activation than checkpoint inhibitors alone (60). Combination approaches that inhibit EV biogenesis, secretion, or uptake, alongside immune checkpoint blockade, could reduce tumor-induced immunosuppression and overcome adaptive resistance, particularly in breast cancers characterized by prominent EV-driven immune exclusion (52). Tumor-derived EVs carrying PD-L1 and other immunosuppressive molecules can sustain T-cell dysfunction despite checkpoint inhibitor treatment, thereby promoting persistent immune escape (11). Accordingly, inhibition of EV production or release has been shown to partially restore T-cell activity and improve responsiveness to checkpoint-targeted therapies by limiting EV-mediated immunosuppressive signaling and reducing T-cell exhaustion (12).

The integration of EV biology into immunotherapeutic strategies may also improve patient stratification and treatment monitoring (128). Circulating EV-associated biomarkers, including EV-PD-L1 and immunoregulatory RNA signatures, may help identify patients most likely to benefit from immune checkpoint inhibitors or combination therapies. Longitudinal monitoring of EV profiles could further facilitate the early detection of therapeutic resistance and support adaptive treatment strategies within precision oncology frameworks (60). Advances in EV engineering and cargo manipulation have expanded the potential of engineered vesicles as therapeutic platforms capable of delivering checkpoint inhibitors, immune-stimulatory molecules, or gene-silencing agents directly to the tumor microenvironment, thereby enhancing therapeutic efficacy while minimizing systemic toxicity (129). Patient-specific EV profiling may additionally enable individualized EV-targeted therapeutic approaches based on distinct EV cargo signatures and associated immune responses (130).

The successful clinical translation of these strategies relies on emerging technologies that enable comprehensive characterization of EV heterogeneity and function (131). High-resolution flow cytometry, single-particle interferometric imaging, advanced microscopy, nano-flow cytometry, ExoView platforms, Raman spectroscopy, and microfluidic technologies now permit detailed single-EV analysis of vesicle size, surface markers, and molecular cargo (132–135). These approaches are particularly valuable for identifying rare but biologically significant EV subpopulations, including PD-L1-positive EVs that may disproportionately contribute to immunosuppression, therapeutic resistance, and disease progression (117, 136). Integration of single-EV profiling with multi-omics technologies and machine learning approaches may further enhance biomarker discovery, patient stratification, and treatment monitoring in breast cancer (137, 138). However, challenges related to assay standardization, reproducibility, analytical complexity, and large-scale clinical validation remain important barriers to routine clinical implementation (139).

Complementary spatial technologies are also providing new insights into EV-mediated communication within the tumor microenvironment. Spatial transcriptomics enables mapping of immune checkpoint gene expression and cytokine signaling across distinct tumor regions, whereas spatial proteomics reveals the localization of immune checkpoint proteins and EV-associated molecules within tissue architecture (140, 141). These approaches are particularly relevant in breast cancer, where the spatial organization of immune cells strongly influences therapeutic response (142). Furthermore, advanced experimental models such as patient-derived organoids and three-dimensional co-culture systems better recapitulate tumor-immune interactions and permit mechanistic investigation of EV-mediated immune regulation under physiologically relevant conditions (143, 144). In parallel, fluorescent and bioluminescent imaging techniques combined with intravital microscopy and whole-body imaging have enabled real-time tracking of EV trafficking and function *in vivo*, providing important insights into pre-metastatic niche formation and immunotherapy responses (145–147). Collectively, these technological advances are expected to accelerate the translation of mechanistic discoveries into clinically effective strategies targeting EV-mediated immune checkpoint regulation in breast cancer (98, 148).

## 9 Challenges, limitations, and knowledge gaps in EV-mediated immune checkpoint regulation

Despite growing evidence supporting the central role of EVs in guiding immune checkpoint signaling in breast cancer, substantial translational barriers remain (52). Explicitly outlining these limitations will be important for placing current findings in context and guiding future EV-based immune-oncology (149). EVs are a heterogeneous family of vesicles in terms of size, origin, cargo, and function (150). Indeed, such heterogeneity has complicated the attribution of specific immunomodulatory effects to particular subsets of EVs, particularly within the TME, where vesicles from tumor cells and surrounding stromal and immune cells coexist (116). The lack of universally accepted markers that define functionally relevant EV subtypes further blocks reproducibility and cross-study comparisons (35). Although international guidelines have improved the reporting of results, inconsistent naming and classification remain a real challenge to standardization (151). There are also technical challenges introduced by isolating and defining EVs that increase complexity (130). The commonly used techniques, such as ultracentrifugation, size-exclusion chromatography, and precipitation methods, have widely varying efficiencies, sample purity, and scalability (152). The co-isolation of EVs with contaminating proteins, lipoproteins, and other cellular components complicates accurate characterization of EV cargo and function, including how much is actually present in EVs and their exact purpose (128). Also, some detection methods lack sufficient sensitivity to detect subsets of EVs, such as those expressing PD-L1.

From a translational research perspective, it remains equally challenging to implement EV-based innovations in clinical settings (153). Preclinical research indicates that inhibiting EV biogenesis, secretion, or endocytosis may enhance immunotherapeutic responses. Nonetheless, concerns regarding specificity and toxicity have been raised (60). On one hand, EVs play critical roles in physiological processes. In this regard, significant inhibition of EV biology may lead to off-target effects (150). On the other hand, innovative therapies such as EVs engineered for drug delivery face challenges associated with large-scale manufacturing and regulatory approval (152). Also, there are no well-established clinical endpoints for EV modulation to ensure translation of laboratory findings to human trials. Taken cumulatively, these points reveal important knowledge gaps in our understanding of EVs and specifically their functions, as well as their relevance in breast cancer immunotherapy (153). These challenges are expected to be addressed through collective efforts to harmonize methodologies and develop more sensitive and specific tools that integrate EV research into properly designed clinical studies.

In conclusion, extracellular vesicles are key regulators of immune checkpoint signaling in breast cancer, facilitating immune evasion, tumor progression, and resistance to immunotherapy by transferring immune checkpoint molecules and immunomodulatory cargo. Their heterogeneous composition across breast cancer subtypes further influences therapeutic responses, highlighting the need for subtype-specific approaches. This review highlights the key role of EVs in mediating communication between tumor, stromal, and immune cells, thereby establishing an immunosuppressive tumor microenvironment that promotes disease progression and therapeutic resistance. Furthermore, it summarizes emerging evidence supporting EV-associated immune checkpoint molecules and regulatory cargo as promising biomarkers and potential therapeutic targets, underscoring their importance in advancing precision immunotherapy for breast cancer.
